# Bay Laurel (*Laurus nobilis* L.) Essential Oil as a Food Preservative Source: Chemistry, Quality Control, Activity Assessment, and Applications to Olive Industry Products

**DOI:** 10.3390/foods11050752

**Published:** 2022-03-04

**Authors:** Stella A. Ordoudi, Maria Papapostolou, Nikolaos Nenadis, Fani Th. Mantzouridou, Maria Z. Tsimidou

**Affiliations:** 1 Laboratory of Food Chemistry and Technology, School of Chemistry, Aristotle University of Thessaloniki, 54124 Thessaloniki, Greece; steord@chem.auth.gr (S.A.O.); papaposm@chem.auth.gr (M.P.); niknen@chem.auth.gr (N.N.); fmantz@chem.auth.gr (F.T.M.); 2 Natural Products Research Center of Excellence (NatPro-AUTH), Center for Interdisciplinary Research and Innovation (CIRI-AUTH), Aristotle University of Thessaloniki, 57001 Thessaloniki, Greece

**Keywords:** *Laurus nobilis* L., bay laurel essential oil, food preservation, flavored olive oil, flavored table olives, Mediterranean diet, low salt formulations, radical scavenging activity, eugenol, methyleugenol

## Abstract

Essential oils (EOs) find application as flavoring agents in the food industry and are also desirable ingredients as they possess preservative properties. The Mediterranean diet involves the use of a lot of herbs and spices and their products (infusions, EOs) as condiments and for the preservation of foods. Application of EOs has the advantage of homogeneous dispersion in comparison with dry leaf use in small pieces or powder. Among them, *Laurus nobilis* (bay laurel) L. EO is an interesting source of volatiles, such as 1,8-cineole and eugenol, which are known for their preservative properties. Its flavor suits cooked red meat, poultry, and fish, as well as vegetarian dishes, according to Mediterranean recipes. The review is focused on its chemistry, quality control aspects, and recent trends in methods of analysis and activity assessment with a focus on potential antioxidant activity and applications to olive industry products. Findings indicate that this EO is not extensively studied in comparison with those from other Mediterranean plants, such as oregano EO. More work is needed to establish authenticity and activity methods, whereas the interest for using it for the preparation of flavored olive oil or for the aromatization and preservation of table oils must be further encouraged.

## 1. Introduction

The Mediterranean diet (MD), which has become part of the intangible UNESCO heritage, is a dietary pattern (a) rich in foods of plant origin (cereals, season fruits vegetables, and legumes, tree nuts, seeds, and olives) (b) moderate to high in the consumption of fish and seafood, and (c) moderate in the intake of eggs, poultry, and dairy products (cheese and yogurt). Consumption of mainly fresh (not cured) red meat used to be rather sporadic and related to important community or family festivities. Virgin olive oil was and still is the main source of added fat in salads and cooked dishes. Spring water was the main drink in a working day. Alcohol consumption (wine and distillates) during meals was also moderate [1]. Seasoning and preservation of foods were widely achieved using local fresh or dried herbs and spices [2]. Modernization and adaptation of the MD to contemporary lifestyle has to consider current food sources availability, global health, food safety directives, and technological innovation. Consumer preference for ‘all natural’ foods and ingredients led to the rise and establishment of functional products in the market [3]. In this view, aromatic and pharmaceutical plants all over the world have been scrutinized as sources of secondary metabolites of proven beneficial health properties [4,5], and are also useful for technological reasons in the food industry due to their flavoring, antioxidant, and preservative properties [2]. In addition, the recognition of virgin olive oil as a functional food not only because of its fatty acid composition but also for the presence of rare bioactive compounds [6] had a positive impact on other products and by-products of the olive industry. For example, table olive manufacturing is reassessed so that the humble food of the poor classes is transformed into a standardized functional industrial product [7].

This review pays attention to the potential of *Laurus nobilis* L. essential oil (EO) as a source of food preservatives for applications to an active Mediterranean food sector, which is the olive industry. Bay laurel, an emblematic plant of the Greek culture, is grown wild or it is cultivated in the Mediterranean basin [8]. Its leaves in dry form and their essential oils are the major commercial products found in the market for food and other applications. The flavor it exerts suits to cooked red meat, poultry, and fish, as well as to vegetarian dishes, according to traditional Mediterranean recipes [9]. Application of EOs in ready-made dishes, bakery products, etc. has the advantage of homogeneous dispersion in comparison with dry leaf use in small pieces or powder [10,11].

The present review focuses on the chemistry, quality control, activity assessment, and current applications of bay laurel EO to the olive industry products.

## 2. The Chemical Composition of *Laurus nobilis* L. Essential Oil

The EO of the dioecious, evergreen plant of *L. nobilis* L. is extracted from the leaves and/or the drupes with common practices such as steam distillation [10].

Bay laurel EO’s main constituents are *α*-pinene, *β*-pinene, sabinene, *α*-terpinene and *γ*-terpinene, eucalyptol (1,8-cineole), linalool, *α*-terpineol and *δ*-terpineol, *α*-terpinyl acetate, eugenol and methyleugenol. Nevertheless, both the total amount (yield) and the composition of the EO derived from different organs of the same plant (stem, leaves, flowers, etc.) seem to differ quantitatively and qualitatively. Table 1 summarizes the results of several publications in which the chemical composition of the EO from various organs of Mediterranean origin bay laurel plants was examined [12,13,14,15].

However, the study of bay laurel EO is not restricted only to material from Mediterranean countries, but it expands to that from other countries [10,16,17,18,19,20,21,22,23,24,25].

Regarding the yield of the EO, this ranges from 1 to 5% when the latter is derived from the fruits of the plant [10] and 0.6 to 1.5% when it comes from the leaves [10,12,13,14,18,19]. The EO yield from the flowers, as well as from other organs or parts of the plant such as bark, wood, stems, buds, and shoots, has been reported to be between 0.2 to 1.2% and not higher than 0.7%, respectively [10,12,18]. Characteristic is the fact that some EO compounds have been identified in almost all studies, irrespective of the plant organ or the geographical origin of the plant material. Table 2 summarizes information about the ranges of percent concentration of the main volatiles of EOs from bay laurel leaves because these are the main source for its commercial production [12,15,16,17,18,19,20,21,22,23,24,25,26,27,28].

Other compounds such as thymol, *p*-cymene, camphene, myrcene, limonene, camphor, spathulenol, *α*-cadinol, as well as some derivatives or isomers of the compounds presented in Table 2, have been also identified. As it can be observed, the concentration range of the individual components of the bay laurel leaf EO is particularly wide even for 1,8-cineole, the characteristic compound of this particular EO. Except for the distillation method [21,22] or the plant material drying method used [20,26], the geographical origin, the harvest season [24,25,29], and the phenological growth stage of the plant [21] seem to contribute to the compositional variation found in the EO of the leaves, though not to the same extent.

**Figure 1:**
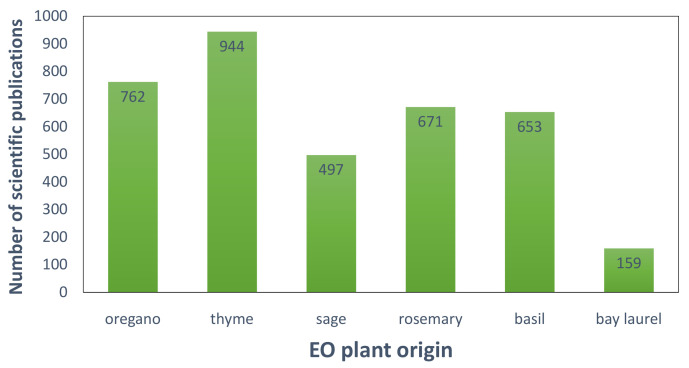
Number of scientific publications for oregano, thyme, sage, rosemary, basil, and bay laurel EOs (retrieved from Scopus on 16 January 2022).

It is noteworthy that in comparison with other species of the Mediterranean flora such as oregano (*Origanum vulgare*), thyme (*Thymus vulgaris*), sage (*Salvia officinalis*), rosemary (*Rosmarinus officinalis*), and basil (*Ocimum basilicum*), the bay laurel EO composition can be considered as understudied. Literature search using “plant common name” OR “plant scientific name” AND “essential oil” AND “composition” in the title, abstract, or keywords, with no time restrictions in the Scopus database (retrieved on 16 January 2022), revealed almost 3 to 6-fold less publications for bay laurel EO in comparison with those for the above-mentioned species (Figure 1).

## 3. Quality and Authenticity Aspects

General quality and authenticity issues are addressed in a plethora of monographs, commercial standards, official guidelines or recommendations for production, and specific regulations for application areas. Except for regulatory authorities, national bodies, international trade standard organizations, pharmacopoeias, industry and consumer associations all over the world cooperate to ensure quality and outline specifications for uses [30,31,32]. Given the increased commercial interest for potential applications of EOs, it is of high priority to tackle issues related to the authentication of the botanical and geographical origin of herbs/spices and their products [31].

Currently, the EO market and e-commerce suffer from illegal practices such as mislabeling and adulteration. Partial substitution by (a) vegetable oils/carriers, alcohols (ethanol), synthetic oils, mineral oils, and (in some cases) water, used as diluents; (b) cheaper EOs from the same species but different geographical origin; (c) cheaper EOs extracted from another organ/part of the plant; (d) cheaper EOs from closely related species; (e) alcohols with high b.p. and (f) pure natural or (semi) synthetic compounds are some of the known fraudulent practices [30,33,34]. Evaluation of the botanical origin of the EOs (species and plant part) is of fundamental importance for their integrity studies and may attract the interest of researchers from plant biology, food, and pharmaceutical fields, which is not usually emphasized in the studies for bay laurel EOs [15].

**Figure 2:**
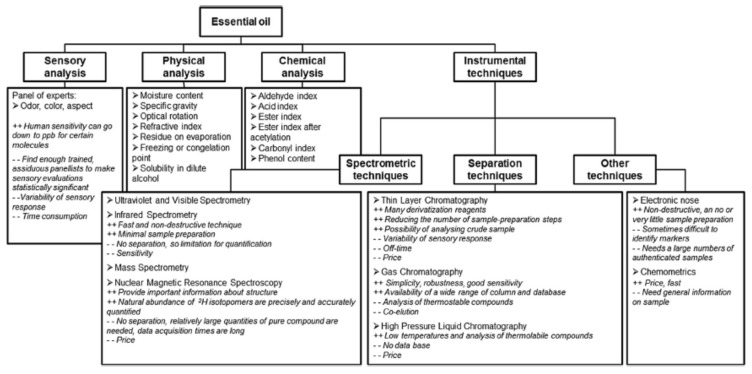
Summary of the existing quality assessment methods of EOs, regarding major advantages (++) and disadvantages (--) (reprinted from [30] with permission from Elsevier).

Quality, especially the certification of EO authenticity, must be ensured through reliable objective methods of analysis. Many reviews and book chapters update the most widely applied quality assessment methods as well as recent analytical advancements [30,35,36,37,38]. An overview of the existing techniques, along with advantages and disadvantages of their application, is illustrated in Figure 2.

**Figure 3:**
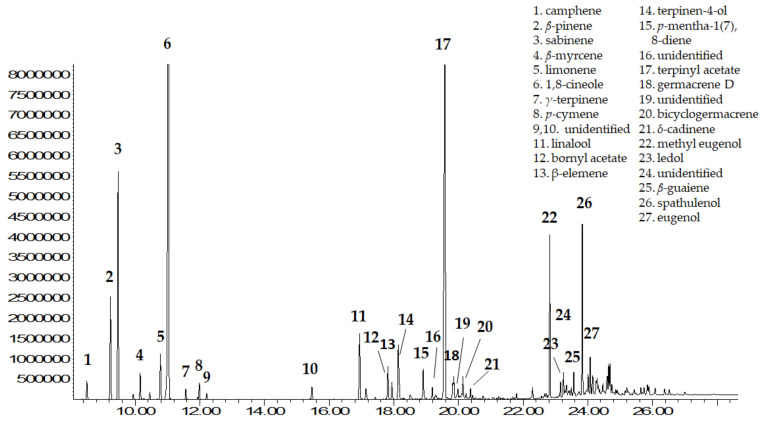
GC-MS chromatographic profile of bay laurel leaf EO under the conditions described by Ordoudi and collaborators [15].

Gas chromatography (GC) coupled with a flame ionization detector (FID) or a mass spectrometer (MS) with a single quadrupole has played a crucial role as a method of choice and is an essential tool in the routine quality control of EOs. Ultrafast separation of EO constituents and fingerprinting/profiling approaches employ more sophisticated MS detectors, i.e., Time-of-Flight (TOF) [36]. The complex composition of the EOs justifies a constant search for new technological solutions in this field. Thus, various analytical protocols are suggested in the relevant literature that may differ according to the characteristics of the capillary columns and the stationary phases as well as the sample preparation method (static or dynamic headspace, solid-phase microextraction, etc). In general, EO analysis is carried out using apolar polysiloxane-based stationary phases, often in combination with moderately polar stationary ones (e.g., polyethylene glycol) to overcome co-elution and to obtain complementary chromatographic data. Simultaneous combination with MS data from in-house and commercial libraries or databases enables component identification [36]. In the case of the bay laurel EO, the existing studies (e.g., [12,15,16,17,18,19,20,21,22,23,24,25,26,27,28]) were carried out under a variety of GC-FID or GC-MS conditions. Figure 3 points up an example of the GC-MS chromatographic profile of the EO from bay laurel leaves that was obtained under the conditions described in [15]. In brief, the protocol involved separation on a DB-WAX capillary column (polyethylene glycol: 30 m × 0.25 mm i.d., 0.33 μm film thickness) and identification using a mass spectrometer operating in the electron impact mode (EI) at 70 eV. Samples were injected manually onto the GC in the split mode at a 25:1 ratio. The volatile constituents were identified by comparing their elution order and mass spectra with data from a commercial library, standards, and published literature.

Very often, compositional characterization of EOs also involves sensory evaluation and basic physicochemical analyses before chromatographic analyses. In search of faster, cheaper, and greener analytical approaches, near-infrared, mid-infrared, and Raman spectroscopy gains importance for a wide range of products, including EOs [39,40]. In particular, the EO spectrum is exploited as the product “fingerprint” for taxonomic specification, quality evaluation, identification, and authentication purposes [38]. Chemometric tools (i.e., exploratory data analysis, data pre-processing, variable selection, pattern-recognition methods) facilitate the analysis of the complex spectroscopic data obtained. Thus, the development of reliable and robust qualitative or quantitative models that can be interoperable and easily accessible is expected to open new perspectives for the quality control of EOs. In their recent review, Kharbach and co-authors [38] summarized the reports published within a timeline of 25 years (1994–2019) about the application of targeted and non-targeted fingerprinting and chemometrics for studying chemotaxonomy of EOs and other product integrity issues (quality, adulteration, geographical/botanical origin). The majority of the 200 studies cited therein refer to targeted metabolite fingerprinting analyses via GC-FID or GC-MS and chemometrics. Most of the rest of the applications involve spectroscopic measurements using Fourier-Transform Mid-Infrared (FT-MIR) and Raman spectrometers. In several cases, where both targeted and non-targeted fingerprinting is carried out, the researchers stress that the results might be equivalent.

Regarding fingerprinting of bay laurel EO, the first relevant study was published recently [41] and was about the development of a Principal Component Analysis (PCA) chemometric model for the discrimination of the plant species based on non-destructive NIR, Raman, and Laser-Induced Breakdown Spectroscopy (LIBS) data. In the same period, Wang et al. [42] reported a rather sophisticated procedure to develop a class model for the prediction of commercial products labeled as “laurel leaves” or “laurel EO” using GC/Q-ToF analytical data and a combination of PCA and Partial Least Squares-Discriminant Analysis (PLS-DA) chemometric methods. More recently, Ordoudi and collaborators [15] presented a less complicated approach to characterize the botanical origin of bay laurel EOs through non-targeted FT-IR fingerprinting and the use of one-class chemometric models (Soft Independent Modeling of Class Analogy, SIMCA). The models were validated against EOs from commercial and botanically certified bay laurel leaves, as well as against a set of commercial EOs from unknown plant origin. Findings were supported by GC-FID/MS analyses. Assignment of the most characteristic bands in the FT-IR transmittance mode, raw and derivative spectra of *L. nobilis* L. leaf EOs was based on data from literature [43,44] and reference compounds [15], and it is presented in Table 3. Such data are useful for researchers who work in the same field.

The region 600–1500 cm^−1^ better describes the skeletal vibrations of the EO constituents and, therefore, it is important for its fingerprint. Discriminating information was derived from variance in the band shape and position within the carbonyl and double bond (1630–1780 cm^−1^) as well as the methyl/methylene group vibrations (e.g., 2800–3100, 1430–1445, and 1360–1380 cm^−1^). Overall, the diagnostic importance of the model was associated with spectral bands at 3060, 1380–1360, 1150, and 1138 cm^−1^. These findings, and previous ones, indicate the importance of FT-IR as a green analytical technique in the quality control of EOs that, as previously mentioned, are frequently subjected to various fraudulent practices (e.g., mislabeling of the botanical origin of the plant material).

## 4. Assessment of the Preservative Properties

EOs are used in the food industry to impart flavor to a wide variety of products. Due to their complex chemical composition, EOs exert biological properties. Some of them, namely antibacterial, antifungal, and antioxidant, are of technological importance, as they can indirectly contribute positively to product preservation [45]. In particular, bay laurel EO contains monoterpene hydrocarbons (*α*- and *β*-pinene, sabinene), oxygenated monoterpene hydrocarbons (1,8-cineol, *α*-terpinyl acetate, linalool, *α*-terpineol), and aromatic compounds (eugenol and methyleugenol) (see Table 2) that can exhibit preservative properties through different mechanisms [46]. The inhibitory effect against foodborne bacteria (such as *Staphylococcus aureus*, *Escherichia coli*, *Listeria monocytogenes* *Salmonella* spp., *Shigella* spp., *Bacillus subtilis*) and fungi belonging to *Aspergillus*, *Fusarium*, *Eurotium*, and *Penicillium* genus are associated with the presence of the two hydrocarbon categories [46,47,48,49,50,51]. These categories are included in the list of flavorings used in foods without restriction, according to EU legislation [52]. Moreover, the antioxidant properties are associated with the presence of aromatic compounds [53,54], among which is eugenol, a known radical scavenger [55]. While eugenol is included in the EU list of permitted flavorings [52], methyleugenol, which is biosynthetically related [56,57] and usually present at higher levels to eugenol, has been reported as toxic, thus restricting its direct application to the food and cosmetics industry [58]. Table 4 incorporates data for the eugenol and methyleugenol concentrations of bay laurel EOs from different Mediterranean countries, which show a considerable natural variability [13,15,18,22,26,27,28,51,53,57,59,60,61,62,63,64,65,66,67,68,69,70,71,72,73,74,75,76,77,78,79,80,81,82,83,84,85,86,87,88,89,90,91,92,93,94,95,96,97,98,99,100].

In particular, methyleugenol is mentioned in both parts (A and B) of Annex III of the Flavoring Regulation (EC) 1334/2008 [101], where it is stated that it should not be added to the food as it is, or, when naturally present in flavorings (such as EOs), should not exceed the maximum values shown in Table 5. Additionally, in the USA, methyleugenol has been removed from the GRAS list by the FDA (Food and Drug Administration) since 2018.

The antimicrobial and antioxidant properties of bay laurel EO are considered well-studied [46,102]. Due to their complex chemical composition, EOs may be effective at different targets in the cell. Their hydrophobic nature favors partitioning in cell membrane lipids and mitochondria. Moreover, EOs seem to act better in environments characterized by low values in pH, temperature, and oxygen concentration.

Efficacy is also affected by the co-presence of compounds that act synergistically/antagonistically [103]. The EO quantities needed are rather low (0.5–20 µL per g of food) and depend on the minimum inhibitory concentration (MIC) values that can be estimated using a number of protocols [104]. In the cited review article, in vitro antimicrobial susceptibility testing methods are presented by principle, together with useful information on their pros and cons. Antioxidant activity may also contribute to the preservative activity of an EO both directly (scavenging of free radicals) and indirectly (via interfering with microbial/fungal metabolism causing oxidation). The in vitro assessment of the antioxidant activity of EOs is usually carried out using the DPPH^•^ (2,2-diphenyl-1-picrylhydrazyl) assay, one of the most widely applied protocol for various types of antioxidants [105]. This is a fact, despite reservations and concerns that have been periodically expressed by scientists on the relevance of the results obtained with this or other exogenous radicals with those from in vivo antioxidant activity studies [55,106]. In this review, focus is given on the assessment of the antioxidant activity of bay laurel. The protocols reported so far for the evaluation of its antioxidant activity present some similarities, but also differences regarding the reaction environment, concentrations of EOs and reagents, time span for reaction monitoring, and expression of results. Table 6 presents a detailed list of the DPPH^•^ conditions of applications to bay laurel EO that can prove useful in future efforts for harmonization and standardization purposes [55,77,89,103,107,108,109,110,111,112,113,114,115,116,117,118].

Most of the cited studies, with some exceptions, confer that the radical scavenging properties are considerable and that bay laurel EO has been found to be more effective than those from some plant species, such as *Myrtus communis*, *Salvia officinalis*, and *Salvia sclarea* [107,116]. The observed activity has been attributed to the presence of 1,8-cineole, eugenol, and methyleugenol. In some cases, literature evidence indicates that 1,8-cineole—the major volatile of this EO—is not active towards DPPH^•^ [119], suggesting a possible contribution due to synergistic effects of compounds when present in a mixture like an EO. To clarify this, and also whether methyleugenol, which does not contain free phenolic groups, contributes to the overall antioxidant activity, Nenadis and collaborators [108] worked with 20 commercial laurel EOs purchased from producers, herbal shops, and pharmacies which were first characterized for their composition using GC-FID/MS and then examined for their DPPH^•^ activity. The results of this study are given in Table 7.

**Figure 4:**
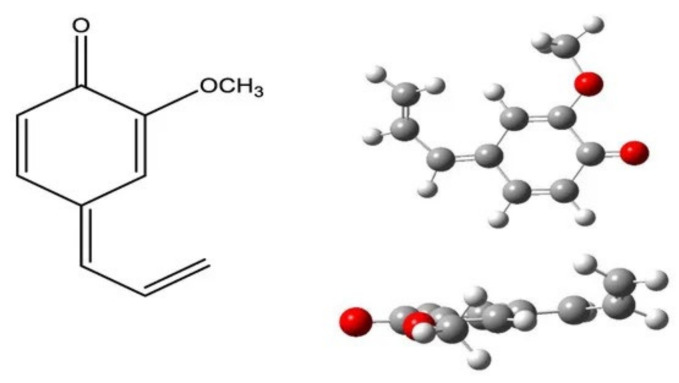
Derived quinone from eugenol after the donation of two hydrogen atoms following a step-wise hydrogen atom transfer (gas phase at B3LYP/6-31G); adopted from Figure 2 in [108]).

The findings indicated a variability in the composition (e.g., 1,8-cineole ranged between 34.1 and 63.0%) and activity (2.1 to 8.1 μmol Trolox/mg EO). The concentration values of the eight major compounds determined, namely *α*-pinene, limonene, 1,8-cineole, linalool, terpinen-4-ol, *α*-terpinyl acetate, methyleugenol, and eugenol, were examined with the aid of Pearson correlation and partial least square regression analyses versus the EO antioxidant activity values. The first approach showed the highest correlation among tested compounds for eugenol (*r* = 0.916, *p* = 0.000), followed by methyleugenol (*r* = 0.785, *p* = 0.000). Other compounds found to contribute less were linalool (*r* = 0.587, *p* = 0.007) and *α*-terpinyl acetate (*r* = 0.484, *p* = 0.031), whereas 1,8-cineole had a negative correlation (*r* = −0.642, *p* = 0.002) with the activity values. Partial Least Square-regression (PLS-R), which takes into account the presence of all the compounds for the formation of a model, verified these observations. Specifically, in the statistically significant model obtained, the values of the standardized coefficients obtained were the highest for eugenol (~2.29), followed by methyleugenol (~0.28). The determination of IC_50_ values for pure compounds showed that methyleugenol had a low activity (IC_50_ = 80 [methyleugenol]/[DPPH^•^], mol/mol) compared with that of eugenol (IC_50_ = 0.3 [eugenol]/[DPPH^•^], mol/mol), whereas linalool and 1,8-cineole were inactive even when tested at high concentration levels. To shed some light on the mechanism of action of methyleugenol, quantum chemical calculations were employed. The outcome of the theoretical calculations suggested the contribution of allyl hydrogens to the activity of methyleugenol. Such hydrogens can be abstracted as easily as the corresponding ones from eugenol in terms of bond dissociation enthalpy values (BDE). However, the superior activity of eugenol was associated with the fact that a hydrogen atom can also be abstracted from the phenolic group, leading to a quinone (Figure 4). For sure, 1,8-cineole was predicted as inefficient considering that BDE values for various C-H bonds were high.

## 5. Applications to Olive Industry Products

Scientific research on applications of EOs in food preservation is increasing following the current interest of the industry and consumers for green solutions. EOs as flavoring agents with preservative properties can be used after direct addition to the food matrix [45,120,121,122], encapsulated [121,123,124,125,126], or after embodiment to packaging materials [121,122,125,126,127,128,129,130,131,132]. The latter two approaches aim at avoiding losses due to volatility and negative reactions of consumers due to the strong flavor at the concentrations necessary to exert preservative effect. On the other hand, encapsulation and incorporation to packaging materials offer the advantage of controlled release of the active compounds over the shelf life of the product, avoiding its over-aromatization.

### 5.1. Applications to Olive Oil Industry

Olive drupes are the source of virgin olive oil, the major fat source in the traditional Mediterranean diet. This oil can be found in two edible categories (‘extra virgin’ and ‘virgin’) in the European Union, the major producer among the Mediterranean countries, as well as in the other non-European Mediterranean producing countries of Maghreb, Jordan, Syria, and Turkey. These countries are also the major consumers of olive oil that is purchased mainly in bulk directly from producers. Bottled virgin olive oil is also consumed in the local market, but is mainly exported to Northern Europe, USA, and Australia. Currently, it also finds its way to the emerging markets of Russia, China, India, and Brazil. Virgin olive oil is the basis for a series of industrial products that are legal blends with refined olive oil, pomace refined oil, and vegetable oils. Flavored olive oils comprise another category of industrial products that has attracted the interest of manufacturers and consumers, as the combinations of flavoring ingredients are unlimited. These oils as commercial products belong to the category “mixed condiments and mixed seasonings” according to the global Harmonised Commodity Description and Coding System (HS Nomenclature). According to Tsimidou [133], ‘flavored’ or ‘aromatized’ olive oils are gourmet products to which the addition of flavoring materials (mainly herbs in dry form or their extracts and EOs) in the production process—or after it—aims at the improvement of sensory characteristics. Nevertheless, as reported in literature [133,134], such an addition can also contribute to improvement of dietary characteristics and increase the shelf life of the end product that can be used as a dressing or for cooking. Production of the innovative industrial flavored oils is based on traditional domestic practices, such as infusion of herbs and dried vegetables in the oil. Systematic knowledge on the benefits and drawbacks of empirical practices should, however, be examined carefully. Commercialization of these products coincided with the interest in the flavors and ingredients of virgin olive oil and herbs such as oregano, rosemary, and other typical plant materials of the Mediterranean diet and cuisine among non-traditional followers [135,136].

For the preparation of infusions, the plant material is grinded and mixed with the oil, the mixture remains for a period defined by experience in the dark or light. It is stirred occasionally, filtered, and transferred to clean glass vessels. The overall process can be accelerated using different means. However, such practices do not always confer desirable attributes to the flavored oils. As reported in the past [137,138], infusions also contain some undesirable compounds that can adversely affect sensory attributes (waxes or bitter compounds) or stability under light exposure (chlorophylls). Moreover, in case when fresh or semi-dried vegetables or herbs are used, there is always a possibility for pathogen development [139]. The addition of essential oils does not encounter such problems. The major restriction is related to the careful choice of the quantities of the added EO or mixtures of EOs so that the end product exerts balanced sensorial characteristics. Bay laurel EO is rarely reported as a flavoring agent of olive oil [140], in contrast to the frequent use of other EOs from the Mediterranean flora [137,140,141,142,143,144,145]. Its addition in olive oil by 0.05% (*v/w*) was found to be promising as far as it concerns 1,8-cineol stability during the thermal oxidation process and under photo-oxidation conditions. Little or no loss of this major constituent was observed [140]. Flavored oils with EOs, herb extracts, or the addition of dry herbs are usually discussed in the literature as end products themselves with reference to their shelf life and sensory characteristics [141,146]. The potential application of a flavored olive oil as a preservation means was only reported once. Indeed, Trabelsi and collaborators [147] prepared flavored olive oils with various herbs or spices, such as *L. nobilis* L., *Rosmarinus officinalis* L., *Zingiber officinale* Roscoe, *Cinnamomum verum* J. Presl, and *Elettaria cardamomum* (L.) Maton, aiming at examining their anisakicidal potency in the anchovy marinating process. Both in vitro and ex vivo assays were performed to meet their purpose. The researchers concluded that the use of flavored olive oils in the industrial marinating process can be considered as an efficient alternative to the freezing process required currently to deactivate *Anisakis* parasite.

Further application of flavored olive oils with EOs is a challenge for those researchers, who examine systematically traditional practices and invest on innovation based on elements of local culture.

### 5.2. Applications to Table Olive Industry

According to the International Olive Council (IOC) [148], “table olive is the product prepared from the sound fruits of varieties of the cultivated olive trees (*Olea europaea* L.) that are chosen for their production of olives whose volume, shape, flesh-to-stone ratio, fine flesh, taste, firmness, and ease of detachment from the stone make them particularly suitable for processing; to remove their bitterness and preserved by natural fermentation; or by heat treatment, with or without the addition of preservatives; packed with or without covering liquid”. The nutritional significance of the end product stems from its high content in monounsaturated fatty acids, vitamin E, and fiber, as well as the presence of numerous phytochemicals [149]. According to IOC statistics, the world production and consumption of table olives has increased steadily since 1990, while from the beginning of the 21st century it doubled, reaching ~2.8 million tons. The European Union contributes with a ~30% share to the world production (data 2021/22). Its production and exports have increased slightly in the last twenty years, while consumption has not changed dramatically (~500–600 thousand tons). Among Mediterranean countries, Spain, Greece, and Italy are the main producers (72,18 and 7% of the total EU production, respectively) (data 2021/22). Egypt (17%) and Turkey (14%) are the other two major producers of table olives (data 2021/22). IOC statistics depict also that low- or even non-producing countries (e.g., United States, Russia, and Brazil) are large consumers of table olives [150].

**Figure 5:**
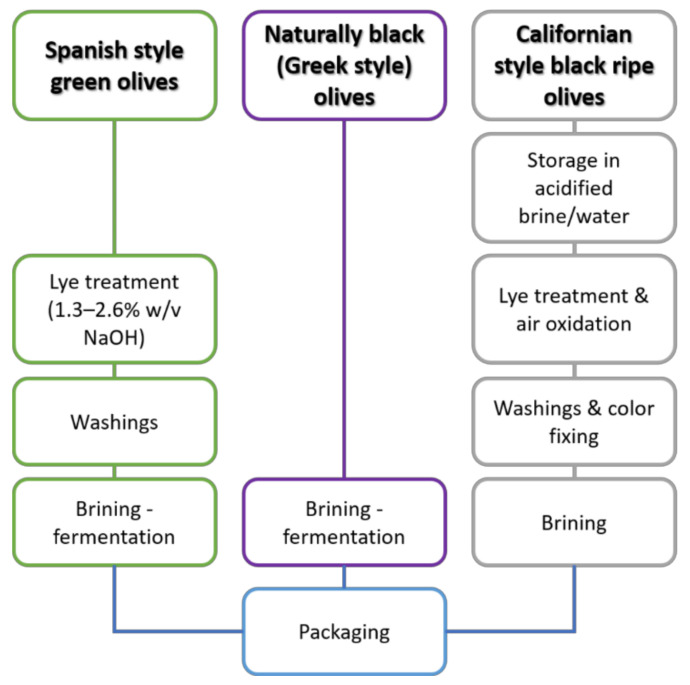
Production process flow charts for Spanish-style green olives, Californian-style black-ripe olives, and naturally black olives in brine.

Figure 5 illustrates the manufacturing process of the most common commercial types of table olives; (1) the Spanish style olives, in which debittering of raw olives takes place by lye treatment (1.3–2.6% NaOH, *w/v*) followed by the removal of excess of lye by repeated washings prior to brining (4–15% NaCl) and fermentation; (2) the naturally black or Greek style olives, in which the debittering step or any other pretreatment is not applied before brining (8–10% NaCl) and fermentation; (3) the Californian style, in which olive fruit polyphenols are oxidized to achieve darkening of the skin. There are also other types of traditionally processed table olives of local interest. The IOC “Trade Standard Applying to Table Olives” describes, in a complete way, all trade and commercial preparations [148].

The majority of fermented or processed olives are stored in bulk in fermentation brine. To boost their economic value, table olives are packed as whole, pitted, and stuffed with various materials (e.g., almonds, anchovies, peppers, and herbs) to add to the sensory attributes or sliced, in glass or plastic containers, tins and plastic, aluminum or polyethylene pouches, filled with brine or gases. With a focus on innovative methods of table olive packaging, modified atmosphere packaging, vacuum packaging, active packaging, edible coating, and film packaging have been proposed to extent the product shelf life [151].

The processing and post-processing technologies for table olives should result in a final product that is microbiologically and chemically safe for the duration of its shelf life and meets consumer’s preferences regarding sensory attributes. It is well documented that salt, along with a low pH environment, protects the product from hazardous and spoilage bacteria. However, when it comes to salt level, conditions of fermentation and preservation in brine do not fully meet safety and nutritional criteria. Nonetheless, table olive processing lowers its nutritional value by decreasing the concentration of bioactive phenolic compounds, especially in the lye treatment and fermentation [152]. As a result, before any attempt to establish table olives as a functional food, this issue must also be addressed.

According to nutritional guidelines for a low-sodium diet [153], the table olive industry is in a transitional phase for the establishment of this traditional fermented food in the Mediterranean area as a healthy product that can be consumed from different age groups with special focus on children, hypertensive consumers, and the elderly. Reformulating processing and preservation methods that use high salinity brine are particularly important. However, this is not a straightforward process, as the role of salt is crucial for the microbiological quality of the end product. *Escherichia coli, Salmonella enterica, Listeria monocytogenes*, and *Staphylococcus aureus* are among the potential foodborne pathogenic bacteria reported in table olive brines [154].

Approaches to innovation primarily concern the use of modified fermentation brines containing less NaCl by means of a mix of other chloride salts (e.g., KCl, CaCl_2_, MgCl_2_) [155,156,157]. However, there is a scarcity of data on the shelf life of low salt table olives and on the solutions that can be offered at a post-fermentation stage. Within the limited available literature [157,158,159], noteworthy findings highlight the advantages of mild preservation techniques (i.e., high hydrostatic pressure and ozone treatments, natural antimicrobials) which have less impact on the nutritional and sensory quality of the final products compared with the traditional techniques (i.e., pasteurization).

Seasoned table olives with dry herbs and spices are commonly found in the markets of the Mediterranean countries or elsewhere. However, there are few scientific works that combine reduction of salt content with the use of herbs, spices, or EOs as a preservation means. This is illustrated in Table 8, which compiles available relevant publications to the best of our knowledge [158,160,161,162].

In particular, Abriouel et al. [158] employed EOs (thyme oil or rosemary oil, 0.2%, *v/v*) in combination with a mixture of condiments (thyme, garlic, and red pepper, 0.5 g/L, each) and application of high hydrostatic pressure for maintenance of desalted Manzanilla Aloreña cracked green table olives in storage brine reduced in salt content. In this study, researchers highlighted the progressive reduction of yeast population caused by rosemary oil during storage, the synergistic bacteriostatic effects of EOs with high hydrostatic pressure treatment, but also the strong impact of EOs on the organoleptic properties of olives. Additionally, Pires-Cabral et al. [161] marked the effectiveness of seasoning Cobrançosa table olives in reduced-salt storage brine with a mixture of thyme (0.06%), oregano (0.04%), calamine (0.02%), garlic (0.6%), and lemon juice (1.2%, *w/w*) in order to mask the bitter taste given by KCl when added to reduced-sodium fermentation brines. However, in the above-mentioned studies, no information is available to document the selection of the plant and EOs types and concentrations chosen.

**Figure 6:**
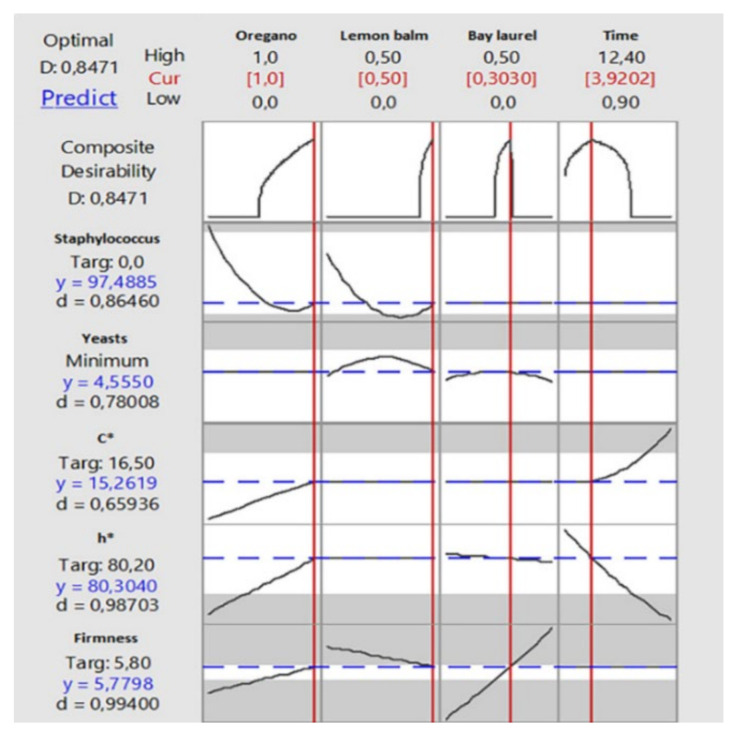
Multiple response optimization plot for growth of *Staphylococcus* (Y_S_) and yeasts (Y_Y_), C * (Y_C_ *), h * (Y_h_ *), and firmness (Y_F_) (data from Figure 6 in [162]).

The only work available for the use of *L. nobilis* EO is that by Papapostolou et al. [162], aiming at the integration of flavored VOO rich in phenolic compounds with EOs from the Greek flora (oregano, lemon balm, and bay laurel) in the traditional manufacturing process of Spanish style green table olives (cv. Chalkidiki) for the production of a tailor-made reduced-salt product. According to the literature, the selected EOs have different antioxidant and antibacterial activities. In particular, oregano EO contains thymol and carvacrol with high antioxidant and antimicrobial activity [163], whereas lemon balm and bay laurel EOs contain a variety of non-phenolic terpenoids that are strong antimicrobials [53,164]. The authors pointed out two critical issues to be considered in similar studies. First, EOs in real food systems may require even 100-fold higher concentrations to be as effective as in vitro models and second, EO effective concentration should not compromise the end product sensory characteristics. Thus, in the above study, the amount of EOs used to make flavored VOO was up to 1% *w/w* (for oregano), 0.5% *w/w* (for lemon balm), and 0.5% *w/w* (for bay laurel), considering both the minimum inhibitory concentration values of each EO for LAB, yeasts, and common pathogens related to table olives (*Escherichia coli*, *Salmonella enterica*, *Listeria monocytogens, Staphylococcus aureus*) as well as the olfactory thresholds of the main EO volatiles. From the results obtained, an optimum oregano, lemon balm, and bay laurel EOs content and storage time combinations of 1.0%, 0.5%, and 0.3%, respectively. Therefore, 3.9 months was proposed to simultaneously reach the target zero value for *Staphylococcus* population (Y_S_), minimize yeast population at an acceptable level, and retain the key quality characteristics of green table olive (color and firmness) (Figure 6).

According to results from a sensory evaluation study (Papapostolou, Mantzouridou, Tsimidou, unpublished data), no preference in color and texture was indicated among samples of reduced sodium table olives stored in VOO without and with the above-mentioned combination of EOs, and the traditional one containing ~4.8 g NaCl/100 g edible flesh. This finding is very important considering that color and texture are the most valued quality characteristics of green table olives from cv Chalkidiki. As regards taste scores, these were lower for the desalted table olives packed in flavored VOO. Noticeably, assessors valued the new table olive product for its “special” taste and flavor but found them quite “strong” and “intense”. Findings seem encouraging toward innovative activities in the medium size local table olive industries based on elements of local culture for the production of tailor-made reduced-salt table olives preserved under mild conditions. The increasing consumer demand for healthy local food products is expected to counteract the shorter shelf life that these products may have. However, it is necessary to educate consumers about the new trends in the industry and also to train them in the sensory characteristics of the new products.

## 6. Conclusions

*L. nobilis* L, known as bay laurel or Apollo laurel, is an evergreen plant, grown wild or cultivated in the Mediterranean countries. Except for the traditional use of leaves as a dry herb, the essential oil contains compounds that have preservative properties of interest to the food industry. Methods for certifying EO integrity are needed beyond legal criteria, whereas its preservative activity is also related to the presence of radical scavengers. Bay laurel EO is understudied in contrast to those of other Mediterranean herbs and spices, such as oregano. Its use per se or in combination with other EOs from the Mediterranean flora is promising for the development of flavored olive oils that can be used not only for salads and cooking but also as preservation means. The example of effective application of bay laurel and other essential oils for the preservation of reduced salt table olives indicates their potential to modernize and add value to traditional products. The olive product industry, which is a key ambassador of the Mediterranean Diet worldwide, looks for innovative solutions that can add further value to traditional products. More research is encouraged in this field that combines science, technology, and gastronomy and meets current nutritional guidelines.

## Figures and Tables

**Table 1:** Chemical composition and percent content of compounds identified in the EO from different organs of Mediterranean origin bay laurel.

Identified Compounds Content (%)	Geographical Origin of the Plant Material	Reference
*Leaf*		
*α*-pinene (2.2), sabinene (4.4), *β*-pinene (1.7), 1,8-cineole (39.1), limonene (2.6), linalool (10.0), terpinen-4-ol (1,4), *α*-terpineol (1.3), *α*-terpinyl acetate (18.2), methyleugenol (11.8), *β*-caryophyllene (1.6) etc.	France	[12]
*α*-pinene (2.8–3.2), sabinene (4.2–4.3), *β*-pinene (2.6–2.7), limonene (1.2), 1,8-cineole (22.8–23.5), linalool (10.6–12.5), terpinen-4-ol (2.6–3.3), *α*-terpineol (3.4–3.9), terpinyl acetate (10.8-11.4), eugenol (1.8–2.6), methyleugenol (8.1–9.4) etc.	Italy	[13]
*α*-pinene (4.3–6.5), *β*-pinene (2.0–4.2), sabinene (9.2–10.2), limonene (0.4–1.1), 1,8-cineole (45.1–53.0), linalool (1.4–3.7), terpinen-4-ol (1.1–2.1), *α*-terpineol (1.2–3.5), terpinyl acetate (11.4–13.1), eugenol (1.2–4.5), methyleugenol (2.3–4.6) etc.	Turkey	[14]
sabinene (4.5–10.6), *β*-pinene (1.8–4.0), limonene (1.3–1.9), 1,8-cineole (26.6–34.9), linalool (1.3–4.1), terpinen-4-ol (1.0–2.8), terpinyl acetate (15.3–31.7), eugenol (0.7–1.8), methyleugenol (1.8–6.4), bornyl acetate (0.7–1.5), spathulenol (1.9–5.3) etc.	Greece	[15]
*Flower*		
1,8-cineole (3.3), (*E*)-ocimene (8.0), terpinyl acetate (2.3), methyleugenol (3.1), *β*-elemene (9.7), *β*-caryophyllene (10.0), *α*-humulene (1.2), germacrene-D 6.1), viridiflorene (12.2), γ-cadinene (4.3), humuladienol (2.3), germacrene-D-4-ol (1.1), viridiflorol (1.0), *β*-eudesmol (2.3), *α*-cadinol (3.4) etc.	France	[12]
*Other plant organs/parts*		
Bark: 1,8-cineole (73.0), terpinen-4-ol (2.3), terpinyl acetate (3.8), *α*-cubebene (1.8), *α*-copaene (1.8), methyleugenol (4.7), *δ*-cadinene (1.0)	France	[12]
Wood: 1,8-cineole (1.6), linalool (3.2), *δ*-terpineol (2.4), terpinen-4-ol (2.4), terpinyl acetate (18.6), eugenol (7.8), *α*-cubebene (1.2), *β*-cubebene (6.0), methyleugenol (16.0), *β*-caryophyllene (1.0), germacrene D (1.2), epicubebol (6.0), *δ*-cadinene (3.6), cubebol (8.1), *β*-eudesmol (3.4), *α*-cadinol (1.1)		

**Table 2:** Summary of the percent content variation of the main volatiles of bay laurel leaf EO irrespective origin ^1^.

Compound	Content (%) ^2^
*Monoterpene hydrocarbons*	
sabinene	0.7–12.2
*α*-pinene	traces–7.7
*β*-pinene	traces–5.0
*α*-terpinene	traces–4.1
γ-terpinene	traces–6.1
*Oxygenated hydrocarbons*	
1,8-cineole	25.7–63.2
linalool	traces–18.5
*α*-terpinyl acetate	traces–27.0
*α*-terpineol	traces–9.3
γ-terpineol	traces–1.9
terpinen-4-ol	traces–6.0
borneol	traces–12.8
*Sesquiterpene hydrocarbons*	
*β*-caryophyllene	traces–1.8
*Phenylpropanoids*	
eugenol	traces–6.5
methyleugenol	traces–21.4

^1^ from [12,15,16,17,18,19,20,21,22,23,24,25,26,27,28]. ^2^ Only compounds in concentrations >1% were included.

**Table 3:** Assignment of the most characteristic bands in the FT-IR transmittance mode spectra of bay laurel leaf EOs based on literature [43,44] and spectra of reference compounds (data from Table 1 in [15]).

Wavenumber (cm^−1^)		Assignment	Relevant Constituent(s)
Zero Order Spectrum	2nd Derivative Spectrum		
Characteristic group vibrations			
3440	-	v_s_(OH)	linalool, terpinene-4-ol, *α*-terpineol
3073; 2985 (sh)	3075; 2986	v_s_(=CH_2_ mono, 1,1) or v_as_(CH_2_) in cyclopropyl rings	methyleugenol *α*-, *β*-pinene, sabinene, spathulenol, linalool, limonene
2965; 2879	2967; 2879–2870	v_as_(CH_3_)	1,8-cineole *α*-, *β*-pinene, sabinene, linalool, terpinene-4-ol
	2947–2945	ν_s_(CH_3_–C=) or (CH_3_)_2_–C–electronegative or (CH_2_) in cyclobutane	1,8-cineole, other unidentified
2925; 2853 (sh)	2924; 2853	v_s_(CH_2_)	sabinene, linalool, *β*-pinene 1,8-cineole
2834 (sh)	2833	(Ar–CH_2_–O) or Ar–OCH_3_	methyleugenol, eugenol
2724	2725	–CHO	unidentified
1730	1732	ν(C=O)	*α*-terpinyl, bornyl, linalyl acetates
	1713–1695	–C=O–OH or aryl–C(H)=O	alkyl ketones (cyclic), aryl aldehydes
1655–1640 (br)	1660–1630	v(C=C) isolated or cyclic	sabinene, linalool, methyleugenol
1514	1516–1514	v(C=C) (ring)	methyleugenol, eugenol, p-cymene
1440–1510	1467–1465	v(C=C–C) (ring) or δ(CH_2_)	methyleugenol, eugenol p-cymene
Skeletal vibrations			
1446	1445;1433	δ_s_(CH_2_) cyclopropyl, cyclobutyl	sabinene, spathulenol, *α*-, *β*-pinene
1375–1363	1377; 1364–1360	v_s_(CH_3_–C=O) δ_s_(CH_3_) gem	1,8-cineole, *α*-terpinyl acetate
1259; 1167–1155	1262–1258; 1155	v_as_(C–O–C) aromatic v_s_(C–O–C) aromatic v(O=C–O)	methyleugenol, eugenol acetate esters
1080	1080	v(C–O–C)	1,8-cineole
1032 (sh)	1033–1031	v_as_(CH_2_–O–C=O)	acetates of primary alcohols
1018	1017		*α*-pinene, γ-terpinene
995	985	δ(C–H)	1,8-cineole
	920–916	(CH_3_)_3_–C–O or 5-membered cyclic ethers	
	887	ω (C–H) γ (=CH_2_)	pinene limonene
	843		
	816	ω (C–H)	p-cymene
	801–797	δ(sp^2^ C–H)	
	770–764	δ(sp^2^ C–H)	

ν, stretching vibration; δ, in plane deformation vibration; γ, out of plane deformation vibration ω, wagging vibration; sh, shoulder; br, broad.

**Table 4:** Eugenol and methyleugenol percent in bay laurel leaf EOs from different Mediterranean countries.

Eugenol	Methyleugenol	Geographical Origin of Plant Material	References (2000–Present)
Content (%)			
trace–2.3	10.6–11.0	Algeria	[59]
2.8	14.0		[60]
2.1	0		[61]
0	2.8		[62]
2.6	4.4		[63]
1.2–3.6	5.1–6.2		[64]
2.4	6.5		[65]
0	16.9		[66]
1.6	7.7	Egypt	[67]
6.4	16.6	France	[60]
0.7–1.8	1.8–6.4	Greece	[15]
12.3	0.9		[68]
9.9	10.4		[69]
0.6–2.2	1.5–6.4		[23]
2.7	3.6		[70]
1.7–6.0	6.9–16.4	Italy	[13]
1.2	4.5		[71]
1.6	3.3		[27]
2.5	10.0	Croatia	[54]
0.2	0.1	Cyprus	[72]
3.7	2.5	Lebanon	[73]
0.6	1.7	Morocco	[74]
1.4	3.9		[62]
5.1	8.7		[75]
2.9	3.5	Turkey	[76]
0.5	0.7		[48]
0–1.7	0–1.1		[77]
trace–0.7	0.4–3.4		[78]
1.1–1.6	1.5–2.1		[79]
trace	3.4		[50]
3.7–4.3	4.6–5.8		[80]
0	0		[81]
1.7	1.5		[82]
0.3	0.2		[83]
0	0		[84]
0.5	0		[85]
0	0.89		[86]
4.2	2.6		[87]
0–2.6	0		[88]
1.3–1.6	2.5–2.8		[89]
0	0		[90]
0.3	0		[91]
0.8	1.4		[92]
0	3.5	Tunisia	[93]
0	3.5		[94]
0.6–4.1	6.6–17.8		[18]
trace–1.6	10.2–10.6		[59]
2.0	13.2		[60]
0.1–5.2	6.20–9.6		[26]
2.1	12.4		[62]
0.5–3.5	6.3–18.8		[28]
1.7–7.2	trace–6.1		[95]
2.2–2.4	15.2–15.6		[96]
0	3.6		[97]
0.4	1.8		[98]
0	11.5		[99]
6.8	4.6		[100]

**Table 5:** Maximum levels of methyleugenol (mg/kg), naturally present in flavorings and food ingredients with flavoring properties, in certain compound food as consumed to which flavorings and/or food ingredients with flavoring properties have been added (abstracted from Annex III, part B in [101]).

Name of the Substance	Compound Food in which the Presence of the Substance is Restricted	Maximum Level (mg/kg)
Methyleugenol	Dairy products	20
	Meat preparations and meat products, including poultry and game	15
	Fish preparations and fish products	10
	Soups and sauces	60
	Ready-to-eat savouries	20
	Non-alcoholic beverages	1

**Table 6:** Brief description of DPPH^•^ protocols used for the evaluation of bay laurel EOs antioxidant activity in chronological order, since 2012.

Reaction Environment	Quantities of EO and Reagents	Reaction End-Point (min)	Reference Compounds	Result Expression	Reference
Ethanol	0.1 mL EO solution (concentration range. n.s.) 2 mL solution DPPH^•^ (0.21 mM)	60	BHT	AAI = DPPH^•^_final concentration_ (μg/mL)/EC_50_ (μg/mL)	[53]
Ethanol	0.3 mL EO solution (30–1000 μg/mL) 1.5 mL solution DPPH^•^ (0.25 mM)	30	-	RSA = [(A_0_–A_1_)/A_0_] × 100	[73]
Methanol	3 mL EO solution (1.25–10 µL/mL) 1 mL solution DPPH^•^ (1 mM)	30	BHT	RSA = [(A_0_–A_1_)/A_0_] × 100	[107]
Methanol	1 mL EO solution (20–2000 μg/mL) 2 mL solution DPPH^•^ (0.1 mM)	60	Quercetin BHT	IC_50_ (μg/mL)	[110]
Ethanol/buffer	0.1 mL EO mixed and vortexed (8 min) with Tris-HCl buffer (pH 7.4) (volume n.s.) 1 mL solution DPPH^•^ (0.5 mM)	20	-	RSA = [(A_0_–A_1_)/A_0_] × 100	[85]
Methanol	0.1 mL EO solution (0.0625–10%. *v/v*) 0.9 mL solution DPPH^•^ (0.07 mM)	30	BHT	RSA = [(A_0_–A_1_)/A_0_] × 100 IC_50_	[111]
Methanol	0.4 mL EO solution (0–4000 μg/mL) 2 mL solution DPPH^•^ (0.06 mM)	30	-	RSA = [(A_0_–A_1_)/A_0_] × 100 IC_50_	[112]
Ethanol	0.05 mL EO solution (0–4000 μg/mL) 5 mL solution DPPH^•^ (0.004%)	30	BHT	IC_50_ (mg/mL)	[113]
Methanol	1 mL EO solution (0.02–0.5 mg/mL) 1 mL solution DPPH^•^ (0.1 mM)	30	Ascorbic acid (0.001–0.2 mg/mL)	IC_50_ (mg/mL)	[99]
Methanol	0.05 mL EO solution (100–1000 μg/mL) 5 mL solution DPPH^•^ (0.004% *w/v*)	30	BHT	IC_50_ (μg/mL)	[114]
Ethanol	0.1 mL EO solution (range of concentration, n.s.) 0.75 mL solution DPPH^•^ (0.1 mΜ)	30	BHT Ascorbic acid	IC_50_ (μg/mL)	[115]
Ethanol	0.01 mL EO 0.5 mL solution DPPH^•^ Adjustment to 1 mL with solvent	15	Trolox	RSA = [(A_0_–A_1_)/A_0_] × 100 TEAC (μg/mL)	[116]
Methanol	0.1 mL EO solution (range of concentration, n.s.) 4 mL solution DPPH^•^ (0.071 mM)	60	Trolox (100–600 μmol/L)	TEAC (mmol/L)	[117]
Methanol	0.1 mL EO solution (12 geometric dilutions in methanol, actual range of concentration, n.s.) 0.1 mL solution DPPH^•^ (0.2 mM)	30	Trolox	IC_50_ (µg/mL) TEAC = IC_50Trolox_ (μΜ)/IC_50sample_ (mg/L)	[118]
Methanol	0.04 mL EO 0.16 mL solution DPPH^•^ (0.1 mM)	30	BHT BHA *α*-Tocopherol	IC_50_ (μg/mL)	[109]
Methanol	0.1 mL EO solution (5 mg/mL) 2.9 mL solution DPPH^•^ (0.1 mM)	60	Trolox, *α*-Tocopherol	μmol Trolox or *α*-Tocopherol/mg EO	[108]

AAI: antioxidant activity index; IC50 (half maximal inhibitory concentration): the concentration of the test compound required for the scavenging of DPPH^•^ by 50%; RSA: radical scavenging activity, where A_0_ the absorbance in the absence of EO (blank) and A_1_ the absorbance in the presence of EO; TEAC: Trolox equivalents; n.s.: not stated.

**Table 7:** Antioxidant activity of bay laurel essential oils (EOs) expressed as Trolox (or *α*-Tocopherol) equivalents and GC-FID-based percent content of their most abundant components ^1^ (abstracted from Table 5 in [108]).

EO	Antioxidant Activity (*n* = 3)	Most Abundant Volatiles							
	μmol Trolox/mg EO (μmol *α-*Tocopherol/mg EO)	*α*-pinene	limonene	1,8-cineole	linalool	terpinen-4-ol	terpinyl acetate	methyleugenol	eugenol
		RI ^1^							
		1043	1120	1159	1456	1584	1683	2082	2338
		*m*/*z* ^2^							
		n.a. ^3^	68,93,136	43,81,154	41,71,153	71,111,154	43,121,181	147,163,178	103,149,164
		Content (%)							
1	4.4 ± 0.0 ^g^ (4.8 ± 0.0 ^g^)	6.0	0.9	61.9	2.8	3.4	9.1	1.1	0.8
2	5.6 ± 0.0 ^h^ (6.0 ± 0.0 ^h^)	7.1	1.6	51.0	3.0	2.8	14.0	1.7	1.2
3	4.7 ± 0.1 ^g^ (5.0 ± 0.1 ^g^)	5.6	2.1	58.4	0.9	2.5	14.2	1.1	0.8
4	8.1 ± 0.1 ^i^ (8.4 ± 0.1 ^i^)	-	-	34.1	10.6	6.1	28.0	7.6	2.1
5	3.6 ± 0.0 ^c^ (4.0 ± 0.0 ^c^)	2.5	3.5	48.6	1.8	4.3	16.1	1.3	1.5
6	3.9 ± 0.2 ^abd^ (4.3 ± 0.2 ^abd^)	7.1	2.9	59.5	3.3	1.6	8.9	2.5	0.5
7	7.7 ± 0.2 ^k^ (8.0 ± 0.2 ^k^)	6.4	2.2	58.2	6.1	1.6	8.8	2.2	2.1
8	3.6 ± 0.1 ^ac^ (4.0 ± 0.1 ^ac^)	7.5	2.6	58.0	3.5	2.2	8.9	2.2	0.5
9	4.0 ± 0.1 ^bd^ (4.4 ± 0.1 ^bd^)	7.5	2.7	59.1	3.5	1.9	8.2	2.2	0.5
10	3.8 ± 0.3 ^abc^ (4.2 ± 0.3 ^abc^)	6.4	2.3	59.6	3.5	2.3	10.2	2.2	0.5
11	4.2 ± 0.2 ^d^ (4.5 ± 0.2 ^d^)	8.2	2.7	54.7	3.6	1.7	10.4	2.6	0.6
12	3.9 ± 0.1 ^abd^ (4.3 ± 0.1 ^abd^)	7.0	2.5	59.1	3.5	1.5	9.7	2.8	0.5
13	3.0 ± 0.1 ^f^ (3.4 ± 0.1 ^f^)	6.7	2.5	63.0	3.8	1.9	9.3	1.0	0.4
14	5.8 ± 0.0 ^h^ (6.1 ± 0.0 ^h^)	2.9	1.3	53.6	6.7	2.8	13.2	5.2	1.2
15	8.1 ± 0.3 ^i^ (8.4 ± 0.3 ^i^)	3.6	2.3	47.7	12.9	1.3	14.0	8.0	2.1
16	2.4 ± 0.0 ^e^ (2.8 ± 0.0 ^e^)	7.4	2.2	61.4	3.6	1.3	10.7	1.1	0.4
17	3.2 ± 0.2 ^f^ (3.7 ± 0.2 ^f^)	8.6	3.1	57.7	3.5	1.6	8.7	2.1	0.4
18	2.5 ± 0.1 ^e^ (2.9 ± 0.1 ^e^)	3.2	1.4	53.2	10.7	-	13.2	-	0.3
19	2.1 ± 0.3 ^j^ (2.6 ± 0.3 ^j^)	3.2	1.6	61.2	1.4	2.4	12.9	0.8	0.3
20	3.7 ± 0.0 ^abc^ (4.1 ± 0.0 ^abc^)	7.1	3.0	58.4	3.3	1.8	8.6	2.5	0.5

^1^ identification based on experimental retention index (RI) determined on a polar TR-FAME column; ^2^ the two qualifier and the molecular ions used for GC-MS identification; ^3^ not applicable as compounds eluting before 7 min were not detected due to solvent delay; column-wise values with different lower-case letters are statistically different at *p* < 0.05.

**Table 8:** Scientific publications including the terms “flavored” or “aromatized” or “aromatised” or “seasoned” and “table olive” in their title, abstract, or keywords, retrieved from Scopus on 3 February 2022.

Type of Table Olives	Condiments	Concentration in Reduced-Sodium Packing Brine (Unless Otherwise Stated)	Aim of the Study	Reference
Seasoned cracked green table olives (cv. Aloreña)	Garlic, pepper, fennel and thyme	4.0% *w/w*	Mineral nutrient content evaluation Sensory evaluation (trained panelists)	[160]
Seasoned cracked green table olives (cv. Aloreña)	Garlic, pepper, fennel and thyme	4.0% *w/w*	Microbial stability Physicochemical stability	[160]
Cracked green table olives (desalted) (cv. Manzanilla Aloreña)	Garlic, pepper, fennel and thyme Thyme or rosemary EOs	0.5 g/L (each) 0.2% (*v/v*)	HHP treatment effect (singly or combined with natural antimicrobials) on microbial stability of olives	[158]
Reduced-sodium table olives (reduced-sodium fermentation brine) (cv. Cobrançosa)	Garlic, lemon juice *Thymus* sp., *Origanum* sp. and *Calamintha nepeta*	0.6% *w/w* 1.2% *w/w* 0.06% *w/w* 0.04% *w/w* 0.02% *w/w*	Nutritional evaluation Safety evaluation Sensory evaluation (trained panelists),	[161]
Reduced-sodium (desalted) Spanish-style green table olives (cv. Chalkidiki)	*Origanum vulgare* ssp. Hirtum EO *Melissa officinalis* EO *Laurus nobilis* EO	0–1% *w/w* (VOO) 0–0.5% *w/w* (VOO) 0–0.5% *w/w* (VOO)	Common pathogens, Fermentation-related microorganisms, Color and texture parameters Nutritional parameters	[162]

HHP: high hydrostatic pressure.

## Data Availability

The data presented in this study are available on reasonable request from the corresponding author.
